# Brain tumor segmentation in multimodal MRI *via* pixel-level and feature-level image fusion

**DOI:** 10.3389/fnins.2022.1000587

**Published:** 2022-09-14

**Authors:** Yu Liu, Fuhao Mu, Yu Shi, Juan Cheng, Chang Li, Xun Chen

**Affiliations:** ^1^Department of Biomedical Engineering, Hefei University of Technology, Hefei, China; ^2^Anhui Province Key Laboratory of Measuring Theory and Precision Instrument, Hefei University of Technology, Hefei, China; ^3^Department of Electronic Engineering and Information Science, University of Science and Technology of China, Hefei, China

**Keywords:** brain tumor segmentation, medical image fusion, pixel-level fusion, feature-level fusion, convolutional neural networks

## Abstract

Brain tumor segmentation in multimodal MRI volumes is of great significance to disease diagnosis, treatment planning, survival prediction and other relevant tasks. However, most existing brain tumor segmentation methods fail to make sufficient use of multimodal information. The most common way is to simply stack the original multimodal images or their low-level features as the model input, and many methods treat each modality data with equal importance to a given segmentation target. In this paper, we introduce multimodal image fusion technique including both pixel-level fusion and feature-level fusion for brain tumor segmentation, aiming to achieve more sufficient and finer utilization of multimodal information. At the pixel level, we present a convolutional network named PIF-Net for 3D MR image fusion to enrich the input modalities of the segmentation model. The fused modalities can strengthen the association among different types of pathological information captured by multiple source modalities, leading to a modality enhancement effect. At the feature level, we design an attention-based modality selection feature fusion (MSFF) module for multimodal feature refinement to address the difference among multiple modalities for a given segmentation target. A two-stage brain tumor segmentation framework is accordingly proposed based on the above components and the popular V-Net model. Experiments are conducted on the BraTS 2019 and BraTS 2020 benchmarks. The results demonstrate that the proposed components on both pixel-level and feature-level fusion can effectively improve the segmentation accuracy of brain tumors.

## 1. Introduction

Automatically and accurately segmenting brain tumor areas from multimodal magnetic resonance imaging (MRI) scans can provide crucial information about tumors including shape, volume, and localization. Based on these information, quantitative assessment of lesions can be carried out, which is of great significance to disease diagnosis, treatment planning, survival prediction, and other relevant tasks. Most existing brain tumor segmentation studies are concentrating on gliomas since they are the most common brain tumors in adults. However, due to the factors like the variety of tumor size, shape and position, the fuzzy boundaries, and the difference in intensity distribution of MRI data obtained by different devices, the accurate segmentation of brain tumors is always a very challenging task (Zhao et al., 2018).

**Figure 1:**
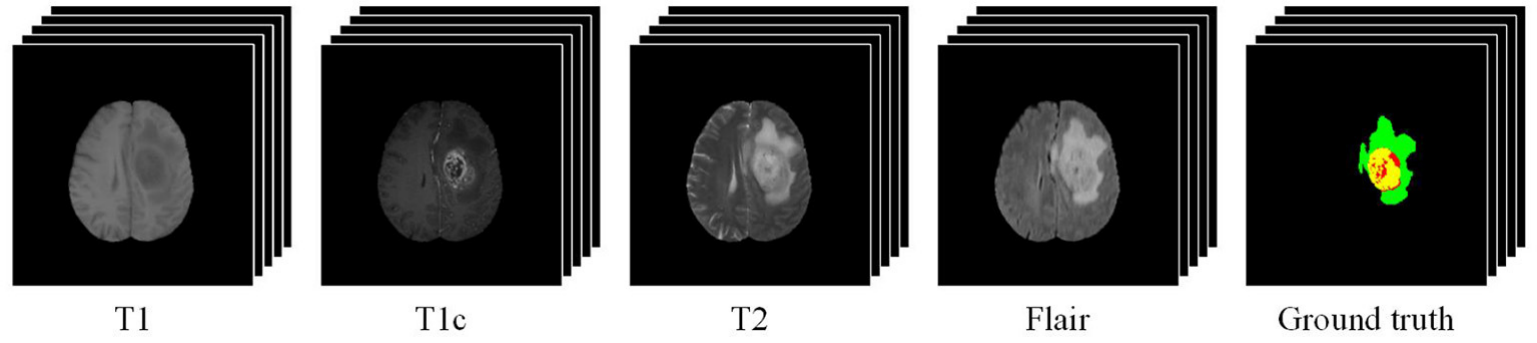
An example of multimodal MRI volumes for brain tumor segmentation. The green, red, and yellow regions in the ground truth indicate edema (ED), non-enhancing tumor and necrosis (NCR/NET), and enhancing tumor (ET), respectively.

Owing to the good ability in capturing high-resolution anatomic structure of tissues, MRI is mostly used in brain tumor segmentation. Commonly-used MRI modalities for brain tumor segmentation include T1-weighted (T1), contrast-enhanced T1-weighted (T1c), T2-weighted (T2), and fluid attenuated inversion recovery (Flair). Figure 1 gives an example of multimodal MRI volumes for brain tumor segmentation, which comes from the dataset released by the Brain Tumor Segmentation (BraTS) challenge (Menze et al., 2015), an annual event held by the Medical Image Computing and Computer Assisted Intervention (MICCAI). The segmentation label (i.e., ground truth) provided by physicians is also shown in Figure 1. The green, red, and yellow regions indicate edema (ED), necrosis and non-enhancing tumor (NCR/NET), and enhancing tumor (ET), respectively. In the BraTS challenge, the segmentation performance is evaluated on three partially overlapping sub-regions of tumors, namely, whole tumor (WT), tumor core (TC), and enhancing tumor (ET). The WT is the union of ED, NCR/NET, and ET, while the TC includes NCR/NET and ET. We can see from Figure 1 that different pathological features of tumors are captured by MRI data of different modalities.

In recent years, various brain tumor segmentation methods have been proposed. Traditional image segmentation methods based on threshold, region, and pixel clustering are difficult to achieve good results in this task due to its high complexity as mentioned above (Liu et al., 2014). The performance of machine learning approaches based on hand-crafted features and classifiers like support vector machines and random forests is still limited in most cases. In the last few years, deep learning-based methods have emerged as the trend in this field due to their obvious advantages on segmentation accuracy (Bakas et al., 2018). Some methods adopt a 2D or 3D patch-based manner, in which convolutional networks are applied to predict the class of the center voxel (Havaei et al., 2017; Kamnitsas et al., 2017; Zhao et al., 2018). However, these methods tend to ignore the correlation among different patches within a large receptive field. To better address the global contextual information, the encoder-decoder architectures represented by U-Net (Ronneberger et al., 2015) and V-Net (Milletari et al., 2016) have become more and more popular in brain tumor segmentation (Wang et al., 2017; Li et al., 2019a; Zhang et al., 2020a; Zhou et al., 2020).

As brain tumor segmentation in MRI is essentially a multimodal image segmentation problem, the joint utilization of multimodal information plays a critical role in this task (Zhang et al., 2022). However, we argue that most existing methods do not pay enough attention to this issue and the utilization of multimodal information is not sufficient. In existing brain tumor segmentation methods, the most common way of using multimodal MR images is to simply stack them or their low-level features as the model input (Cao et al., 2021; Chen et al., 2021; Valanarasu et al., 2021; Wang et al., 2021; Zhang et al., 2021b). In addition, as mentioned above, MR images with different modalities reflect different pathological features (Chen et al., 2021; Wang et al., 2021), so their importance to a given segmentation target should be different. However, many methods fail to take this difference into consideration in their segmentation models and there is a lack of refinement for multimodal features, which will have an adverse effect on the segmentation performance.

In this paper, we address the above problems *via* the multimodal image fusion technique at both the pixel level and the feature level. For one thing, we adopt pixel-level image fusion to enrich the input modalities of the segmentation model and the fused modalities can strengthen the association among different types of pathological information captured by multiple source modalities. For another, we embed an attention-based feature fusion module into the segmentation network to refine multimodal features for better segmentation performance. Specifically, the main contributions of this work are summarized into four points:

1. To make use of multimodal information more sufficiently for brain tumor segmentation, we introduce the multimodal image fusion technique including both pixel-level fusion and feature-level fusion into the segmentation task.
2. We present a pixel-level image fusion network (PIF-Net) to fuse 3D multimodal MR images, aiming to enrich the input modalities of the segmentation model. This is actually a modality enhancement approach since the fused modalities obtained by the PIF-Net can effectively combine the pathological information from multiple source modalities.
3. To address the difference among multiple modalities for a given segmentation target, we design an attention-based modality selection feature fusion (MSFF) module for multimodal feature refinement and it is embedded into the segmentation network for performance improvement.
4. We propose a two-stage brain tumor segmentation framework based on the PIF-Net, the MSFF module and the V-Net. Experimental results on the BraTS 2019 and BraTS 2020 benchmarks demonstrate the effectiveness of the proposed pixel-level and feature-level fusion approaches for brain tumor segmentation.

The rest of this paper is organized as follows. Section 2 introduces the related works. In Section 3, the proposed method is presented in detail. The experimental results and discussion are given in Section 4. Finally, we conclude the paper in Section 5.

## 2. Related work

### 2.1. Brain tumor segmentation

Many automatic brain tumor segmentation methods have been proposed in recent years. They can be roughly divided into two categories (Havaei et al., 2017): the generative model-based methods and the discriminative model-based methods. The generative model-based methods require domain-specific prior knowledge about the appearance characteristics of tumorous and healthy tissues, but they are challenging to characterize due to the complexity of brain tissues. The discriminative model-based methods treat brain tumor segmentation as a pattern classification problem for the voxels in MRI volumes and they have become the mainstream in this field owing to the rapid development of machine learning techniques. Popular hand-crafted features used in brain tumor segmentation include local histograms (Goetz et al., 2014), structure tensor eigenvalues (Kleesiek et al., 2014), texture features (Subbanna et al., 2013), and so on, while typical shallow learning models such as support vector machines and random forests are frequently adopted in brain tumor segmentation (Bauer et al., 2011; Meier et al., 2014; Pinto et al., 2015).

In the last few years, deep learning has rapidly achieved the dominance in brain tumor segmentation owing to the significantly improved performance. Some early methods adopt a patch-based classification manner by utilizing convolutional networks to predict the class of the center voxel of a 2D or 3D image patch. Havaei et al. (2017) proposed a two-pathway architecture to extract features with 2D convolutional kernels of different sizes. They also explored three cascade architectures in which the output of the first network with larger input size is supplemented as an additional source for the second network to extract information of multiple scales simultaneously. The DeepMedic (Kamnitsas et al., 2017), a well-known 3D brain tumor segmentation model proposed by Kamnitsas et al., also adopts a dual pathway architecture that uses patches of different sizes as the network input, aiming to incorporate both local and larger contextual information. In addition, the dense training scheme is employed in Kamnitsas et al. (2017) to address the relationship among neighboring patches. Zhao et al. (2018) integrated fully convolutional neural networks (FCNNs) and the conditional random field (CRF) into a unified framework for brain tumor segmentation. In their method, features are also extracted from receptive fields of different sizes.

The above patch-based classification methods can't fully consider the correlation among neighboring patches and the range of the receptive field is always limited, although some improved strategies are adopted. To address this problem, the encoder-decoder semantic segmentation architectures such as U-Net (Ronneberger et al., 2015), 3D U-Net (Çiçek et al., 2016), and V-Net (Milletari et al., 2016) have become more and more popular in brain tumor segmentation. Myronenko (2018) proposed a segmentation method that won the first place in the BraTS 2018 challenge by adding an variational auto-encoder (VAE) branch into an encoder-decoder architecture to obtain an additional regularization to the encoder part. To alleviate the issue of class imbalance, some methods apply a cascaded architecture to decompose the original multi-label segmentation problem into multiple binary segmentation sub-problems. Wang et al. (2017) cascaded three CNNs to realize the segmentation of three tumor areas including WT, TC and ET. Zhang et al. (2020a) proposed a task-structured brain tumor segmentation network to address the task-modality and task-task relationship simultaneously. Zhou et al. (2020) proposed a one-pass multi-task network with cross-task guided attention for brain tumor segmentation, which integrates the multiple segmentation sub-tasks into one deep model. Li et al. (2019a) proposed a multi-step cascaded network that takes the hierarchical topology of the brain tumor sub-structures into account and segments the sub-structures from coarse to fine.

However, it is worth noting that current study on brain tumor segmentation does not pay enough attention to the joint utilization of multimodal MR images, which is in fact a key issue in this multimodal image segmentation task (Zhang et al., 2022). The most common way of using multimodal MR images is to simply stack them or their low-level features as the model input (Cao et al., 2021; Chen et al., 2021; Valanarasu et al., 2021; Wang et al., 2021; Zhang et al., 2021b). In addition, many methods treat each modality data with equal importance to a given segmentation target (Chen et al., 2021; Wang et al., 2021). These factors motivate us to introduce image fusion technique including both pixel-level fusion and feature-level fusion into the brain tumor segmentation framework for better performance.

### 2.2. Pixel-level medical image fusion

The purpose of pixel-level medical image fusion is to integrate the complementary information contained in multimodal medical images by generating a composite fused image, which is expected to be more suitable for human or machine perception. A variety of medical image fusion methods have been proposed over the past few decades and most of them are developed under a “decomposition-fusion-reconstruction” three-phase framework (Li et al., 2017; Liu et al., 2020b). Specifically, the source images are first decomposed into a transform domain and the decomposed coefficients from different source images are then fused. The fused image is finally reconstructed based on the fused coefficients. Multi-scale transform (MST) and sparse representation (SR) are two main categories of image decomposition that are widely used in medical image fusion (Liu et al., 2015, 2016, 2019, 2021; Du et al., 2016; Yang et al., 2016; Li et al., 2017; Zhang et al., 2018; Zhu et al., 2018; Yin et al., 2019).

However, most previous works in medical image fusion focus on the 2D image fusion problem, while methods for 3D image fusion were rarely studied (Yin, 2018). Using 2D fusion methods to tackle 3D medical images slice by slice independently neglects the correlation among adjacent slices and thereby tends to lose spatial contextual information of volumetric data. Wang et al. (2014) proposed a 3D multimodal medical image fusion method based on the 3D discrete shearlet transform (3D-DST) and designed a global-to-local strategy to fuse the decomposed coefficients. Yin (2018) introduced the tensor sparse representation (TSR), which is a high-dimensional extension of 2D SR, for 3D medical image fusion. Nevertheless, in these methods, the source images are treated equally in the fusion framework with identical decomposition approach and isotropic fusion strategy. As a result, the characteristics of different source modalities are not fully considered, leading to the loss of important modality information.

Recently, deep learning has emerged as an active direction in the field of image fusion (Liu et al., 2018; Zhang et al., 2021a) and some medical image fusion methods based on deep learning models like CNNs and generative adversarial networks (GANs) have been proposed (Liu et al., 2017, 2022; Liang et al., 2019; Ma et al., 2020a, 2022; Zhang et al., 2020b; Tang et al., 2021; Xu and Ma, 2021; Xu et al., 2022). By optimizing the loss functions that are specially designed based on the characteristics of source modalities, the deep learning-based methods have advantages over conventional MST-based and SR-based fusion methods on preserving modality information. However, the above deep learning-based methods are generally developed for 2D image fusion. In this work, we present a CNN-based 3D medical image fusion approach and introduce it for brain tumor segmentation by enriching the input modalities. In fact, current study on pixel-level medical image fusion is mostly devoted to pursuing good visual quality for physician observation and high evaluation results on objective metrics of image fusion, while very few study focuses on the application of image fusion to some specific clinical machine vision problems such as classification, detection and segmentation. Therefore, this work is also of high significance from the viewpoint of medical image fusion.

## 3. The proposed method

### 3.1. Overview

**Figure 2:**
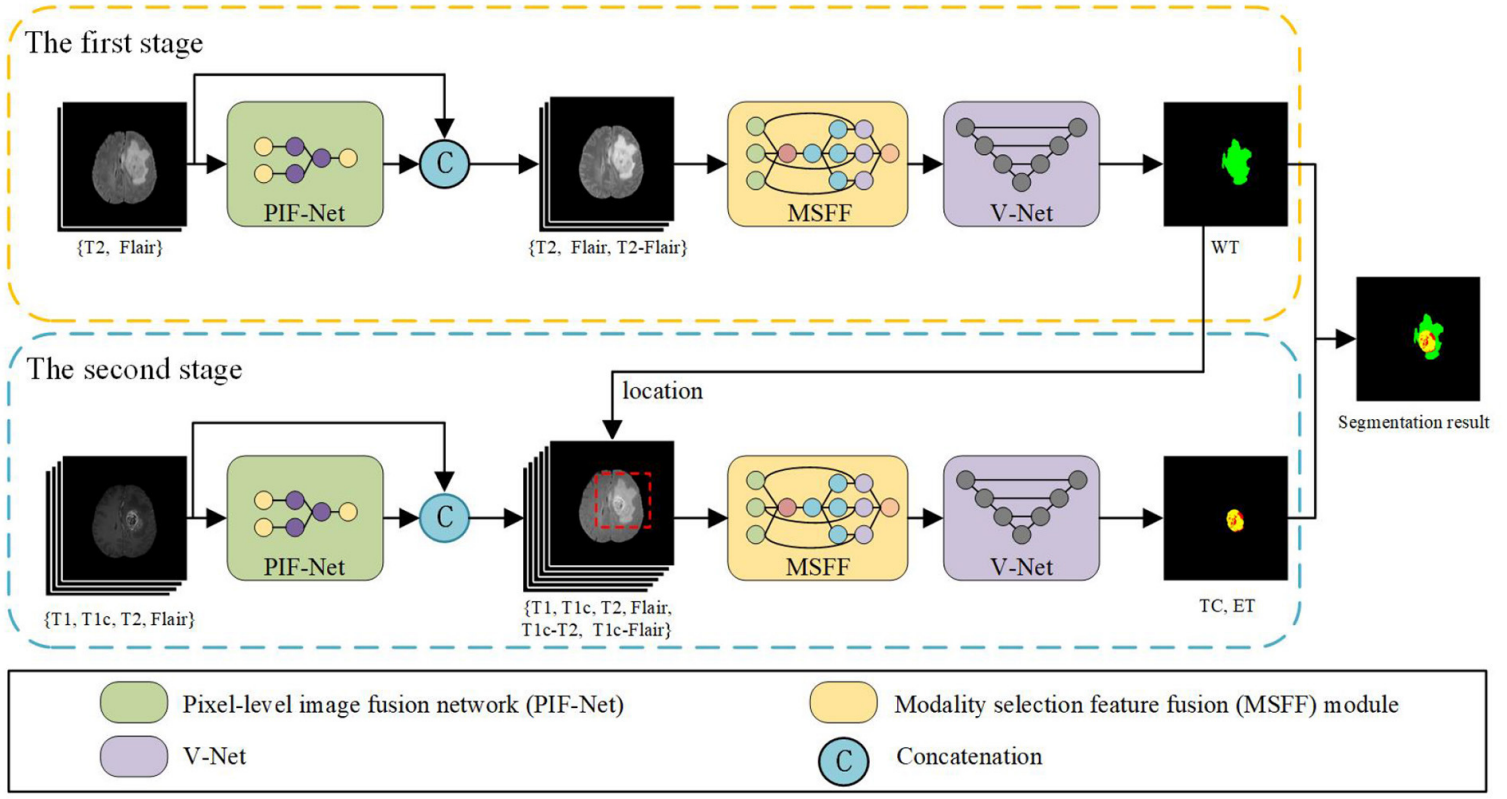
The schematic diagram of the proposed brain tumor segmentation framework.

Figure 2 shows the schematic diagram of the proposed brain tumor segmentation framework. It consists of two stages to achieve the segmentation result of WT, TC, and ET areas. The two stages share a similar architecture that is composed of a PIF-Net to enrich the input modalities of the segmentation model *via* pixel-level fusion, an MSFF module to refine the mutlimodal features *via* feature-level fusion, and a V-Net (Milletari et al., 2016) with the encoder-decoder structure to obtain the segmentation result. The target of the first stage is to segment the WT area, while the second stage aims to identify the TC and ET areas. Since the TC and ET areas are included in the WT area, the segmentation result of the first stage is used to locate the input region of the second stage, which is helpful to alleviate the class imbalance issue. The sliding window-based approach introduced in Lyu and Shu (2020) is adopted to determine the input region of the second stage, namely, the window that contains the maximum number of tumor voxels is selected. In addition, considering that the peritumoral edema are mainly highlighted in T2 and Flair modalities, we only use T2 and Flair as the input source modalities in the first stage. The PIF-Net is used to generate their fused modality, which is denoted as T2-Flair. These three modalities (i.e., T2, Flair and T2-Flair) are fed together to the subsequent MSFF module in the first stage. In the second stage, all the four source modalities (i.e., T1, T1c, T2, and Flair) are adopted as the original input. The PIF-Net is applied to obtain two additional fused modalities, which are the fusion of T1c and T2 (denoted as T1c-T2), and the fusion of T1c and Flair (denoted as T1c-Flair). We mainly choose the T1c modality for fusion because it is known to be very effective in detecting the TC and ET areas. By contrast, the T1 modality provides relatively less information for segmenting brain tumors and it generally plays an auxiliary role in this task (Bakas et al., 2018; Ma and Yang, 2018). Thus, the input of the MSFF module in the second stage contains six modalities in total. The final segmentation result is achieved by combining the results obtained at two stages together.

### 3.2. PIF-Net

**Figure 3:**
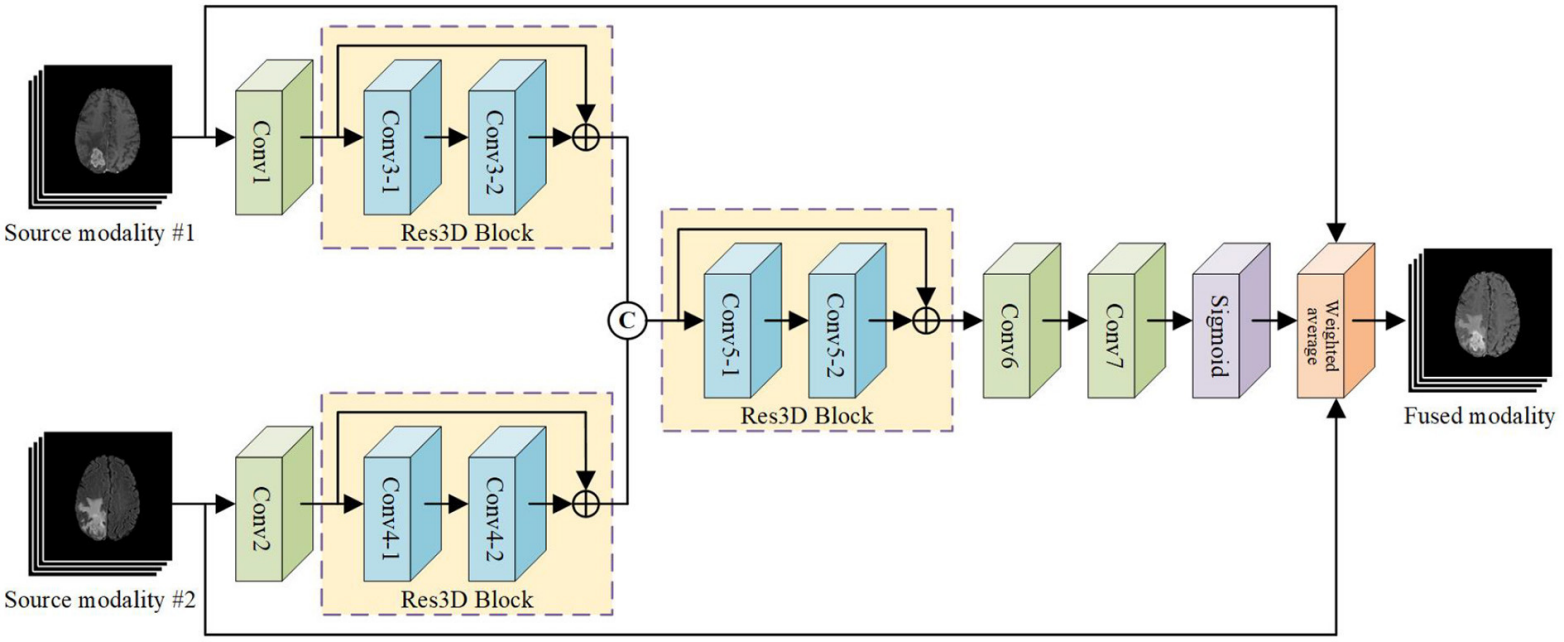
The architecture of our PIF-Net for 3D multimodal MR image fusion.

Considering the high computational cost and memory usage of 3D convolutional networks, we design a relatively plain network architecture as shown in Figure 3 for 3D pixel-level image fusion. Note that this is likely to be the first work on CNN-based 3D medical image fusion to our knowledge, as mentioned in Section 2.2. The PIF-Net contains two branches for feature extraction from two source modalities. Each branch is composed of a 3 × 3 × 3 convolutional layer and a 3D residual (denoted as Res3D) block that contains two 3 × 3 × 3 convolutional layers using the skip connection. The feature maps obtained from two branches are then concatenated and fed to another Res3D block. Two 3 × 3 × 3 convolutional layers are further applied to reduce the number of channels to 1 and a sigmoid operation is conducted to reconstruct a weight mask. Finally, the fused modality is reconstructed by performing the weighted average calculation based on the mask and source images. It is worth noting that the fused image can also be reconstructed directly from the fused feature maps without using a weight mask. However, since the voxels in the meaningless background regions have zero-valued intensity in each source modality, a direct regression tends to cause inappropriate non-zero predictions in these regions, which will affect the fusion quality. The voxel-wise weighted average strategy adopted can effectively avoid this problem and we experimentally found that it can produce good fusion results. The detailed parameter configuration of the network architecture is given in Table 1.

**Table 1:** Detailed parameter configuration of the PIF-Net.

**Layer**	***K*_*s*_**	***S*_*s*_**	***P*_*s*_**	***I*_*c*_**	***O*_*c*_**	***A***
Conv1	3 × 3 × 3	1	1	1	32	ReLU
Conv2	3 × 3 × 3	1	1	1	32	ReLU
Conv3-1	3 × 3 × 3	1	1	32	32	ReLU
Conv3-2	3 × 3 × 3	1	1	32	32	/
Addition	/	/	/	32	32	ReLU
Conv4-1	3 × 3 × 3	1	1	32	32	ReLU
Conv4-2	3 × 3 × 3	1	1	32	32	/
Addition	/	/	/	32	32	ReLU
Conv5-1	3 × 3 × 3	1	1	64	64	ReLU
Conv5-2	3 × 3 × 3	1	1	64	64	/
Addition	/	/	/	64	64	ReLU
Conv6	3 × 3 × 3	1	1	64	32	/
Conv7	3 × 3 × 3	1	1	32	1	/
Sigmoid	/	/	/	1	1	/
Weighted average	/	/	/	1	1	/

*K*_*s*_, *S*_*s*_, *P*_*s*_, *I*_*c*_, *O*_*c*_, and *A* denote the kernel size, stride, padding size, number of input channels, number of output channels, and activation operation, respectively.

The definition of loss function is a key issue in deep learning-based image fusion methods as it determines the preservation of modality information from source images. In this work, the loss function of our PIF-Net is formulated as


(1) $$\begin{array}{l} L_{pif}=L_{pixel}+\alpha L_{ssim}, \end{array}$$

where *L*_*pixel*_ and *L*_*ssim*_ indicate the pixel loss and the structural similarity loss, respectively. α is the regularization parameter that balances these two terms, and it is experimentally set to 450 in our method. The pixel loss is designed to preserve the intensity information, which is often related to the lesions(e.g., edema) that have very high or low intensity in some MRI modalities. It is defined as


(2) $$\begin{array}{l} L_{pixel}=||\text{F}-{\text{S}}_{1}{||}_{F}^{2}+\beta ||\text{F}-{\text{S}}_{2}{||}_{F}^{2}, \end{array}$$

where **S**_1_ and **S**_2_ denote the source images, and **F** denotes the fused image. β is the trade-off parameter and $||\cdot |{|}_{F}^{2}$ denotes the tensor Frobenius norm. The structural similarity loss is adopted to extract anatomic structure information from source images and it is defined as


(3) $$\begin{array}{l} L_{ssim}=\gamma \left(1-\text{SSIM}\left(\text{F},{\text{S}}_{1}\right)\right)+\left(1-\text{SSIM}\left(\text{F},{\text{S}}_{2}\right)\right), \end{array}$$

where SSIM(·, ·) represents the 3D structural similarity measure and γ is the trade-off parameter.

The parameters β and γ are set according to the specific characteristics of fusion problems. In the first stage, for the fusion of T2 and Flair images, β and γ are both set to 1 since these two modalities have relatively similar pathological and structural information. In the second stage, let **S**_1_ and **S**_2_ denote the T1c and T2/Flair images, respectively. Considering that the T2/Flair image contains more lesion information regarding the edema area, we increase the weight of T2/Flair images in *L*_*pixel*_. Meanwhile, since the T1c image captures more tissue structures in the TC and ET areas, a larger weight is assigned to the T1c image in *L*_*ssim*_. In our method, we set both β and γ to 2 for the fusion of T1c and T2/Flair images.

**Figure 4:**
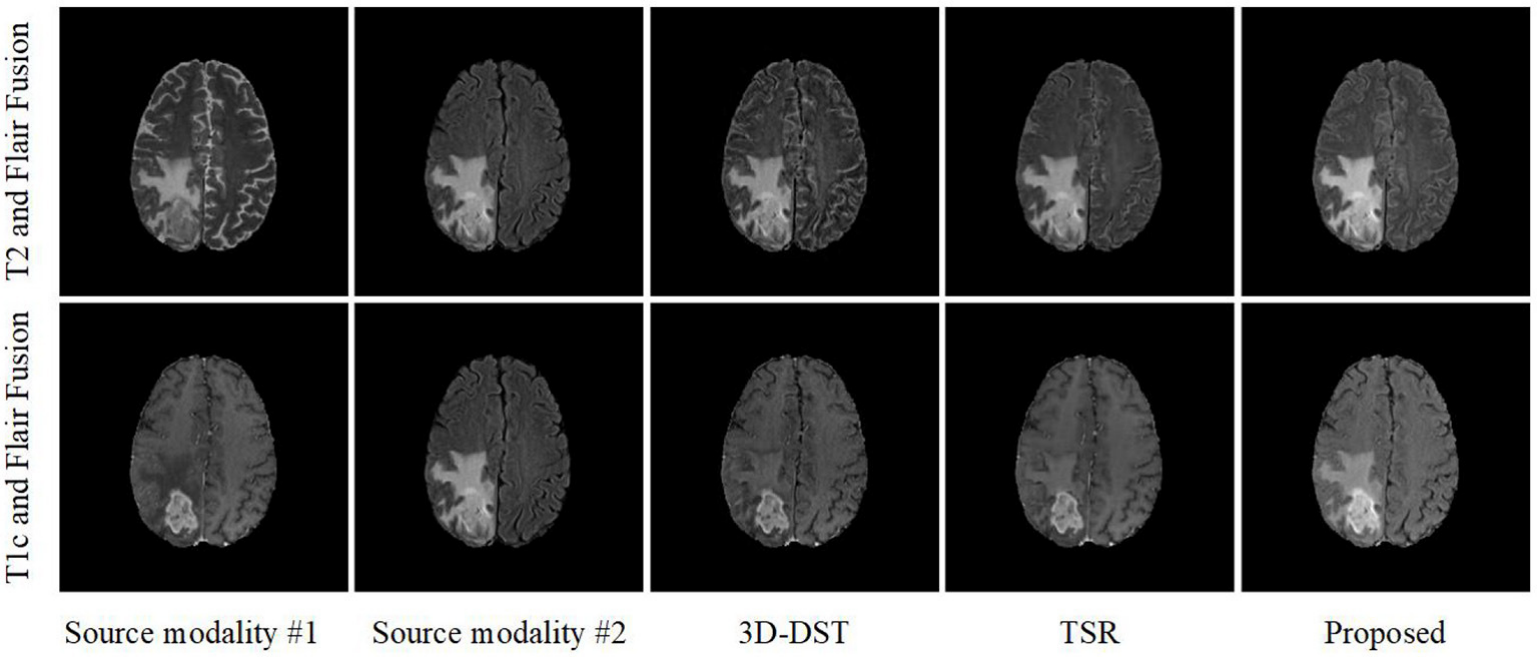
An example of fusion results obtained by different 3D medical image fusion methods.

The PIF-Net is trained based on the training set released by the BraTS challenge 2019. The training set contains 335 cases of multimodal MRI volumes and four modalities (i.e., T1, T1c, T2, and Flair) are provided in each case. The original volumes of size 155 × 240 × 240 are cropped into patches of size 80 × 80 × 80 by the sliding window technique to enlarge the scale of the training set. The learning rate is fixed as 10^−4^ during the training process and the Adam optimizer is adopted to train the network. Figure 4 shows an example of fusion results obtained by the PIF-Net. The results of two representative 3D medical image fusion methods 3D-DST (Wang et al., 2014) and TSR (Yin, 2018) are also provided for comparison. The results of T2 and Flair fusion and T1c and Flair fusion are given at the first and second rows in Figure 4, respectively. It can be seen that the PIF-Net achieves higher fusion quality than the other two methods on the tumor areas, especially for the T1c and Flair fusion, in which the 3D-DST and TSR methods fail in preserving the edema information contained in the Flair images well, while the PIF-Net simultaneously preserve important modality information from both two source images.

### 3.3. MSFF module

**Figure 5:**
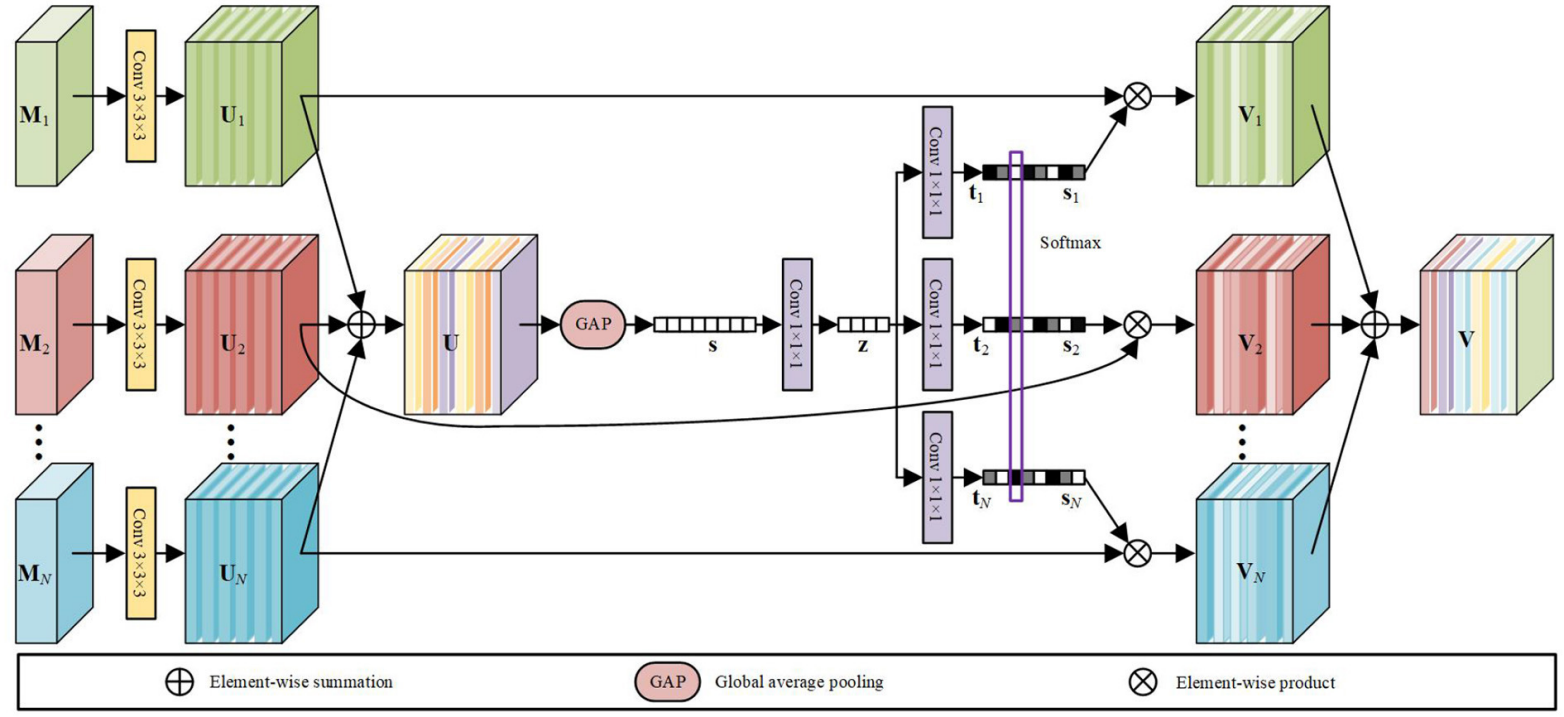
The architecture of our MSFF module for multimodal feature refinement.

The MSFF module is designed to refine the features extracted from multimodal MRI volumes for subsequent segmentation. Inspired the selective kernel network (SKNet) for multi-scale feature extraction (Li et al., 2019b), an attention-based feature fusion module is presented to adaptively adjust the weights of the features from different modalities. The architecture of our MSFF module is shown in Figure 5. Let ${\text{M}}_{1},{\text{M}}_{2},\ldots ,{\text{M}}_{N}\in {\mathbb{R}}^{L\times H\times W\times 1}$ denote the input multimodal MRI volumes that involve both the original source modalities and the fused modalities obtained by the PIF-Net, where *N* is total number of input modalities. A 3 × 3 × 3 convolutional layer is firstly performed on each input volume for feature extraction. The obtained features are denoted as ${\text{U}}_{1},{\text{U}}_{2},\ldots ,{\text{U}}_{N}\in {\mathbb{R}}^{L\times H\times W\times C}$, where *L* × *H* × *W* denotes the size of the 3D feature map and *C* denotes the number of feature maps. In our method, *C* is set to 16. The features from different sources are firstly merged *via* an element-wise summation as


(4) $$\begin{array}{l} \text{U}=\overset{N}{\underset{i=1}{\sum }}{\text{U}}_{i}. \end{array}$$

Then, we embed the global information by a channel-wise global average pooling (GAP) operation to get a feature vector **s** ∈ ℝ^1×1×1×*C*^. Specifically, the *c*-th element of *s* is calculated as


(5) $$\begin{array}{l} s_{c}=\Phi_{GAP}\left({\text{U}}_{c}\right)=\frac{1}{L\times H\times W}\overset{L}{\underset{i=1}{\sum }}\overset{H}{\underset{j=1}{\sum }}\overset{W}{\underset{k=1}{\sum }}{\text{U}}_{c}\left(i,j,k\right). \end{array}$$

Further, a compact feature **z** ∈ ℝ^1×1×1×*C*/*r*^ is generated by a 1 × 1 × 1 convolutional layer for channel reduction, which is actually equivalent to a fully connected layer. The ratio factor *r* is set to 4 in our model. Next, we adopt *N* parallel channel up-scaling convolutions with kernel size of 1 × 1 × 1 to reconstruct *N* *C*-dimensional vectors ${\text{t}}_{1},{\text{t}}_{2},\ldots ,{\text{t}}_{N}\in {\mathbb{R}}^{1\times 1\times 1\times C}$. This is actually the excitation operation used in the SENet (Hu et al., 2018). Subsequently, a channel-wise softmax calculation is performed on each element across all the *N* vectors (indicated by the purple frame) to obtain the attention vectors ${\text{s}}_{1},{\text{s}}_{2},\ldots ,{\text{s}}_{N}\in {\mathbb{R}}^{1\times 1\times 1\times C}$. Specifically, the *c*-th element of *s*_*i*_ is calculated as


(6) $$\begin{array}{l} s_{i,c}=\frac{e^{t_{i,c}}}{\overset{N}{\underset{j=1}{\sum }}e^{t_{j,c}}}, \end{array}$$

where *t*_*i, c*_ denotes the *c*-th element of **t**_*i*_, *i* ∈ {1, 2, …, *N*}, *c* ∈ {1, 2, …, *C*}.

Finally, the fused feature **V** ∈ ℝ^*L*×*H*×*W*×*C*^ is calculated by a channel-wise weighted average over the source features using the attention weights as


(7) $$\begin{array}{l} \text{V}=\overset{N}{\underset{i=1}{\sum }}{\text{s}}_{i}\cdot {\text{U}}_{i}. \end{array}$$

According to a recent survey on attention mechanism (Guo et al., 2022), the attention mechanism used in our MSFF module belongs to the branch attention, which can be viewed as a dynamic branch selection mechanism and typically used in a multi-branch architecture. In the proposed method, to be more specific, the attention mechanism can be regarded as a kind of modality attention, aiming to extract features from multimodal MR images more effectively.

### 3.4. Segmentation loss

The loss function used for training the segmentation model is defined as


(8) $$\begin{array}{l} L_{seg}=L_{dice}+\lambda L_{bce}, \end{array}$$

where *L*_*dice*_ and *L*_*bce*_ denote the dice loss and the binary cross entropy (BCE) loss, respectively, as


(9) $$\begin{array}{l} L_{dice}=1-\frac{2\overset{N}{\underset{i=1}{\sum }}p_{i}g_{i}}{\overset{N}{\underset{i=1}{\sum }}p_{i}^{2}+\overset{N}{\underset{i=1}{\sum }}g_{i}^{2}+\varepsilon }, \end{array}$$

(10) $$\begin{array}{l} L_{bce}=-\frac{1}{N}\overset{N}{\underset{i=1}{\sum }}\left[g_{i}\log p_{i}+\left(1-g_{i}\right)\log\left(1-p_{i}\right)\right], \end{array}$$

where *g*_*i*_ ∈ *G* is the ground truth binary volume, *p*_*i*_ ∈ *P* is the network prediction, and *N* denotes the number of voxels. The parameter ε is a small constant to avoid dividing by 0. The Dice loss is known to be capable of alleviating the class imbalance issue (Milletari et al., 2016), while the BCE is the mostly used loss function for binary classification or segmentation. In brain tumor segmentation, the union of these two losses is a common way as it can combine their complementary advantages. The parameter λ controls the trade-off between these two losses and it is experimentally set to 0.5 in our method.

## 4. Experimental results and discussion

### 4.1. Data and implementation details

The BraTS 2019 and BraTS 2020 benchmarks (Menze et al., 2015) are adopted to demonstrate the effectiveness of the proposed method. The multimodal MRI data in a BraTS benchmark is divided into three parts: a training set, a validation set and a testing set. Only the training set releases the segmentation label (i.e., ground truth) annotated by experts to the public. The validation set is used to adjust model training and the MRI data is available, but the label is not provided. Users must upload their segmentation results to the organizer's sever at https://ipp.cbica.upenn.edu/ to obtain the evaluation results. Both data and label in the testing set are not available to users. In our experiments, just as most previous studies in this field, we adopt the training set for model training and validation, while use the validation set for performance evaluation. In particular, the BraTS training set is further divided into two parts: 80% samples are used for network training and the remaining 20% samples are used as a validation set to guide the training process. The BraTS 2019 training dataset includes 335 cases, while BraTS 2020 has a larger one comprising 369 cases. These multimodal MRI data have been skull-striped, re-sampled, and co-registered. Each case contains MRI data of four modalities (i.e., T1, T1c, T2, and Flair) and each volume is of size 155 × 240 × 240.

For data pre-processing and augmentation, the popular z-score normalization approach is applied to each MRI volume, namely, the data is subtracted by the mean and divided by the standard deviation of the non-zero region. The training volume is randomly cropped into patches of size 128 × 192 × 160 before fed to the network in the first stage. For each volume, the patch of size 128 × 128 × 128 that contains maximum tumor voxels is used for training in the second stage. Moreover, in both two stages, the intensity of each volume is randomly shifted by a value in [−0.1σ, 0.1σ] (σ denotes the standard deviation) and randomly scaled by a factor in [0.9, 1.1]. In addition, a random flipping along each axis is applied with a probability of 50%.

Our network is implemented in PyTorch and trained on two NVIDIA TITAN RTX GPUs. The Adam optimizer is used for updating weights. The learning rate is progressively decayed using the following rule:


(11) $$\begin{array}{l} l=l_{0}\times {\left(1-\frac{i}{N}\right)}^{0.9}, \end{array}$$

where *l*_0_ is the initial learning rate, *i* is an epoch counter and *N* is the total number of the epochs. We experimentally set *l*_0_ to 10^−4^ and *N* to 300.

The labels provided by the BraTS benchmark include the ED, NCR/NET and ET, while the evaluation of segmentation accuracy is performed on three partially overlapping regions: WT (ET + NCR/NET + ED), TC (ET + NCR/NET) and ET, as mentioned in Section 1. In our experiments, we adopt the region-based training strategy, which directly optimizes these three sub-regions instead of individual labels, since its effectiveness has been widely verified in brain tumor segmentation (Isensee et al., 2020). For post-processing, we also adopt a frequently-used approach that the ET is replaced by the NCR/NET when its volume is less than 500 voxels to remove possible false predictions on ET (Isensee et al., 2020; Lyu and Shu, 2020; Zhang et al., 2020a). Two popular objective metrics including the Dice score and the Hausdorff distance (%95) are used to evaluate the segmentation accuracy.

### 4.2. Parameter analysis

**Figure 6:**
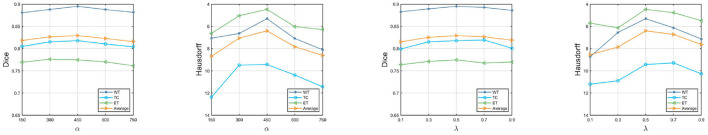
Impact of the parameters α and λ on the model performance.

The loss functions in our method contain several trade-off parameters such as α, β, γ, and λ. The principle for determining the values of β and γ has been detailed in Section 3.2. In this subsection, we analyze the effect of the parameters α and λ on the segmentation performance of the proposed method. The parameter α is used to balance the pixel loss and the structural similarity loss, and these two terms should have relatively close values so that both of them can have sufficient contribution. Based on the experimental observations, we set α to 150, 300, 450, 600, and 750 to study its impact. The corresponding results are shown in the first two sub-figures in Figure 6. It can be seen that the proposed method can obtain relatively stable performance when α is set between 150 and 750, and in particular between 300 and 600. Based on these results, we set α to 450 by default in our experiments. The parameter λ controls the balance between the dice loss and the BCE loss in the segmentation model. Similarly, we set λ to 0.1, 0.3, 0.5, 0.7, 0.9 to analyze its effect on the model performance. The corresponding results are given in the last two sub-figures in Figure 6. We can see that the setting of 0.5 can result in the best performance in most cases, so the parameter λ is set to 0.5 by default in our method.

### 4.3. Ablation study of the proposed method

In this subsection, an ablation study is conducted to evaluate the effectiveness of our PIF-Net and MSFF module in the proposed method. Specifically, the following four models are considered in this study:

- **OURS w/o PIF-Net&MSFF**: Removing the PIF-Net and the MSFF module simultaneously from the proposed brain tumor segmentation framework. In each stage, only the V-Net is remained for segmentation. This is the original baseline for our method.
- **OURS w/o PIF-Net**: Removing the PIF-Net from the proposed brain tumor segmentation framework. The MSFF module is embedded before the V-Net to realize multimodal feature refinement for segmentation in both stages.
- **OURS w/o MSFF**: Removing the MSFF module from the proposed brain tumor segmentation framework. The PIF-Net is used to generate the fused modalities as the additional input of the segmentation model in both stages.
- **OURS**: The complete model proposed in this work.

**Figure 7:**
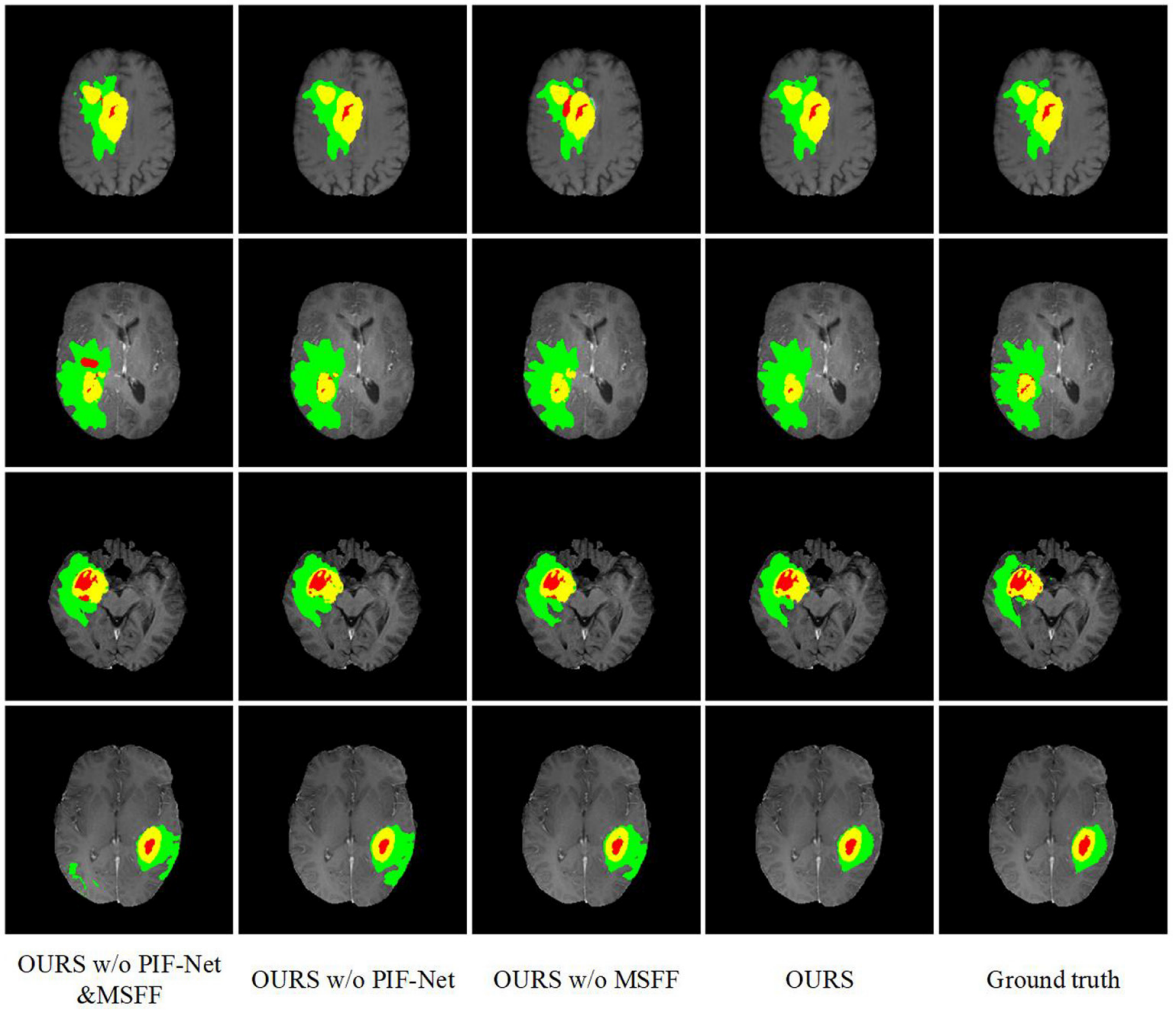
Examples of brain tumor segmentation results obtained by di3fferent methods in the ablation study. The green, red, and yellow regions indicate edema (ED), non-enhancing tumor and necrosis (NCR/NET), and enhancing tumor (ET), respectively.

The evaluation results on the BraTS 2019 and BraTS 2020 benchmarks are listed in Tables 2, 3, respectively. method generally has a better a slightly better performance for BraTS 2020 than performance for BraTS 2020 than BraTS 2019, which is mainly because the BraTS 2020 benchmark contains more training samples in the training set, with additional 34 samples in comparison to the BraTS 2019 benchmark. The comparison between **OURS** and **OURS w/o PIFnet&MSFF** demonstrates that the utilization of our PIF-Net and MSFF module can significantly improve the performance (1.8% to 2.3% in terms of the mean Dice score, and 3.3 to 3.7 in terms of the mean Hausdorff distance) over the baseline model. The comparison between **OURS w/o MSFF** and **OURS w/o PIF-Net&MSFF** (as well as the comparison between **OURS** and **OURS w/o PIF-Net**) verifies the effectiveness of the PIF-Net in improving the segmentation accuracy. The comparison between **OURS w/o PIF-Net** and **OURS w/o PIF-Net&MSFF** (as well as the comparison between **OURS** and **OURS w/o MSFF**) shows that the MSFF module is also beneficial for the segmentation performance. Some segmentation results obtained by **OURS w/o PIF-Net&MSFF**, **OURS w/o PIF-Net**, **OURS w/o MSFF**, and **OURS** are visualized in Figure 7. It can be seen that the complete model can generally obtain more accurate segmentation results than the baseline methods when compared to the ground truth.

**Table 2:** Objective evaluation results for the ablation study of the proposed method on the BraTS 2019 validation sets.

**Tumor region**	**Metrics**	**OURS w/o PIFnet & MSFF**	**OURS w/o PIFnet**	**OURS w/o MSFF**	**OURS**
WT	Dice	0.8635	0.8771	0.8832	**0.8942**
	Hausdorff	7.1211	7.7784	7.1654	**5.3490**
TC	Dice	0.7788	0.8065	0.8045	**0.8142**
	Hausdorff	15.7345	**10.1822**	14.4599	10.8988
ET	Dice	0.7682	0.7698	0.7692	**0.7710**
	Hausdorff	9.1385	**5.3155**	6.4719	5.8548
Average	Dice	0.8035	0.8178	0.8190	**0.8265**
	Hausdorff	10.6647	7.7587	9.3657	**7.3675**

Bold values indicate the best-performing scores on each metric (each row in the tables) among all the four models.

**Table 3:** Objective evaluation results for the ablation study of the proposed method on the BraTS 2020 validation sets.

**Tumor region**	**Metrics**	**OURS w/o PIFnet & MSFF**	**OURS w/o PIFnet**	**OURS w/o MSFF**	**OURS**
WT	Dice	0.8678	0.8725	0.8878	**0.8950**
	Hausdorff	11.5732	9.6274	7.8896	**5.3117**
TC	Dice	0.8025	0.8153	0.8139	**0.8178**
	Hausdorff	11.6728	10.4340	10.9337	**9.4285**
ET	Dice	0.7631	0.7730	0.7678	**0.7745**
	Hausdorff	6.9469	5.9442	7.1674	**4.4715**
Average	Dice	0.8111	0.8203	0.8232	**0.8291**
	Hausdorff	10.0643	8.6685	8.6636	**6.4039**

Bold values indicate the best-performing scores on each metric (each row in the tables) among all the four models.

An interesting observation we can obtain from Tables 2, 3 are that the improvements achieved by the PIF-Net and the MSFF module have their characteristics on different sub-regions. Specifically, for the WT area, the PIF-Net is more effective in improving the segmentation accuracy than the MSFF module. On the other hand, for the TC and ET areas, the MSFF module is more helpful in comparison to the PIF-Net. This phenomenon can be observed from the comparison between **OURS w/o PIF-Net** and **OURS w/o MSFF**. The results shown in Figure 7 also verify this point. By referring to the ground truth, we can see that **OURS w/o MSFF** generally obtains more accurate results for the ED area (shown in green) than **OURS w/o PIF-Net**, while **OURS w/o PIF-Net** performs better for the NCR/NET and ET areas (shown in red and yellow). We provide an explanation to this observation as follows. The segmentation of WT is mainly based on the ED area that can be effectively captured in the T2 and Flair volumes. The modality characteristics on the ED area in T2 and Flair volumes are generally close, so the requirement of multimodal feature fusion or selection is not very urgent. By contrast, the pixel-level image fusion achieved by the PIF-Net can enrich the input modalities for the segmentation model and this modality enhancement approach can also be viewed as a data augmentation method to some extent, which tends to be relatively more effective for WT segmentation as only two source modalities are used. In comparison to WT, the segmentation of TC and ET is more difficult due to the factors like smaller size, more irregular shape, etc. As a result, more modalities are typically required in TC and ET segmentation. In such a situation, the refinement of multimodal features achieved by the MSFF module is of higher significance. Therefore, the segmentation of TC and ET benefits more from the MSFF module. Nevertheless, it is worth noting that our PIF-Net and MSFF module both improve the segmentation accuracy of all the three sub-regions, just with different extents.

### 4.4. Comparison with other methods

In this subsection, we compare the proposed method with some existing brain tumor segmentation methods, which are mainly included in the proceedings of BraTS 2019-2021 challenges and generally have good performance. Tables 4, 5 report the evaluation results of different methods on BraTS 2019 and BraTS 2020 validation sets, respectively. For the comparison methods, the results reported in the original publications are adopted since the benchmarks used are exactly the same. In addition, the results obtained by a single model instead of multi-model ensemble are used for the sake of fair comparison. In each case, the best score is indicated in bold and the second best score is underlined. We can observe from Tables 4, 5 that the proposed method achieves very competitive performance among all the methods. For WT and TC regions, the proposed method obtains the highest Dice scores on both BraTS 2019 and BraTS 2020 validation sets. Our method achieves 0.8265 and 0.8291 in terms of the mean Dice score on these two datasets, which are both in the second place among all the methods. It is worth mentioning that the performance of proposed method may be slightly inferior to some latest state-of-the-art methods. However, the main purpose of this work is to verify the effectiveness of the proposed pixel-level and feature-level image fusion approaches for brain tumor segmentation. The segmentation model and loss function adopted in this work are both plain while popular approaches (i.e., the original V-Net and the BCE-and-Dice-based loss) in 3D medical image segmentation. By introducing some advanced architectures and loss functions, we believe that the segmentation performance can be further improved.

**Table 4:** Objective evaluation results of different brain tumor segmentation methods on the BraTS 2019 validation sets.

**References**	**WT**		**TC**		**ET**		**Average**	
	**Dice**	**Hausdorff**	**Dice**	**Hausdorff**	**Dice**	**Hausdorff**	**Dice**	**Hausdorff**
Xu et al. (2019)	0.8930	6.9640	0.8070	7.6630	0.7590	**4.1930**	0.8197	**6.2733**
Baid et al. (2019)	0.8700	13.3600	0.7700	12.7100	0.7000	6.4500	0.7800	10.8400
González et al. (2019)	0.8882	8.1231	0.7833	7.5618	0.7231	4.9132	0.7982	6.8660
Lorenzo et al. (2019)	0.8904	-	0.7511	-	0.6634	-	0.7683	-
Ahmad et al. (2019)	0.8518	9.0083	0.7576	10.6744	0.6230	8.4683	0.7441	9.3837
Abraham and Khan (2019)	0.8605	-	0.7108	-	0.6323	-	0.7345	-
Bhalerao and Thakur (2019)	0.8527	8.0793	0.7091	9.5708	0.6668	7.2700	0.7429	8.3067
Yan et al. (2019)	0.8600	40.3100	0.7300	10.4000	0.6600	18.5300	0.7500	23.0800
Iantsen et al. (2019)	0.8700	8.3500	0.7900	9.5800	0.6700	7.8200	0.7767	8.5833
Astaraki et al. (2019)	0.8700	5.9000	0.8100	**7.1600**	0.7100	6.0200	0.7967	6.3600
Cao et al. (2021)	0.8938	7.5050	0.7875	9.2600	**0.7849**	6.9250	0.8221	7.8967
Wang et al. (2021)	0.8889	7.5990	0.8141	7.5840	0.7836	5.9080	**0.8289**	7.0303
Valanarasu et al. (2021)	0.8760	8.9420	0.7392	9.8930	0.7321	6.3230	0.7824	8.3860
OURS	**0.8942**	**5.3490**	**0.8142**	10.8988	0.7710	5.8548	0.8265	7.3675

Bold and underlined values indicate the best scores and second best scores on each metric (each column in the tables) among all the methods.

**Table 5:** Objective evaluation results of different brain tumor segmentation methods on the BraTS 2020 validation sets.

**References**	**WT**		**TC**		**ET**		**Average**	
	**Dice**	**Hausdorff**	**Dice**	**Hausdorff**	**Dice**	**Hausdorff**	**Dice**	**Hausdorff**
Jun et al. (2020)	0.8780	6.3000	0.7790	11.0200	0.7520	30.6500	0.8030	15.9900
Liu et al. (2020a)	0.8823	6.4900	0.8012	**6.6800**	0.7637	21.3900	0.8157	11.5200
Messaoudi et al. (2020)	0.8413	-	0.6804	-	0.6537	-	0.7251	-
Sun et al. (2020)	0.8920	-	0.7880	-	0.7230	-	0.8010	-
Cirillo et al. (2020)	0.8926	6.3900	0.7919	14.0700	0.7504	36.0000	0.8116	18.8200
Pang et al. (2020)	0.8811	18.0901	0.7605	29.0570	0.7538	34.2391	0.7985	27.1287
Sundaresan et al. (2020)	0.8900	**4.4000**	0.7700	15.3000	0.7700	29.4000	0.8100	16.3667
Ballestar and Vilaplana (2020)	0.8300	12.3400	0.7700	13.1100	0.7200	37.4200	0.7733	20.9567
McHugh et al. (2020)	0.8810	6.7200	0.7890	10.2000	0.7120	40.6000	0.7940	19.1733
Ma et al. (2020b)	0.8794	-	0.7731	-	0.7040	-	0.7855	-
Cao et al. (2021)	0.8934	7.855	0.7760	14.5940	**0.7895**	11.0050	0.8196	11.1513
Wang et al. (2021)	0.8900	6.4690	0.8136	10.4680	0.7850	16.7160	**0.8295**	11.2177
Zhang et al. (2021b)	0.8800	6.9500	0.7400	30.1800	0.7000	38.6000	0.7733	25.2433
OURS	**0.8950**	5.3117	**0.8178**	9.4285	0.7745	**4.4715**	0.8291	**6.4039**

Bold and underlined values indicate the best scores and second best scores on each metric (each column in the tables) among all the methods.

## 5. Conclusion

In this paper, we mainly introduce pixel-level and feature-level image fusion techniques for MRI-based brain tumor segmentation, aiming to achieve more sufficient and finer utilization of multimodal information. Specifically, we present a CNN-based 3D pixel-level image fusion network named PIF-Net to enrich the input modalities of the segmentation model and design an attention-based feature fusion module named MSFF for multimodal feature refinement. A two-stage brain tumor segmentation framework is accordingly proposed based on the PIF-Net, the MSFF module and the V-Net. Experimental results on the BraTS 2019 and BraTS 2020 benchmarks show that the proposed components on both pixel-level and feature-level fusion can effectively improve the segmentation accuracy of all the three tumor sub-regions including whole tumor, tumor core and enhancing tumor. The pixel-level image fusion network in this work is trained independently to the segmentation model. Future work may concentrate on integrating image fusion and segmentation into a unified network for better feature learning to further improve the segmentation performance.

## Data availability statement

The datasets for this study can be found in the BraTS 2019 dataset available at: https://www.med.upenn.edu/cbica/brats2019/data.html and in the BraTS 2020 dataset available at: https://www.med.upenn.edu/cbica/brats2020/data.html.

## Author contributions

YL: conceptualization, methodology, and writing. FM: methodology, experiments, and Writing. YS: methodology and experiments. JC: methodology and review. CL: experiments and review. XC: methodology, review, and supervision. All authors contributed to the work and approved the submission.

## Funding

This work was supported in part by the National Natural Science Foundation of China under Grants 62176081, 61922075, 62171176, and 41901350, and in part by the Fundamental Research Funds for the Central Universities under Grants JZ2020HGPA0111 and JZ2021HGPA0061.

## Conflict of interest

The authors declare that the research was conducted in the absence of any commercial or financial relationships that could be construed as a potential conflict of interest.

## Publisher's note

All claims expressed in this article are solely those of the authors and do not necessarily represent those of their affiliated organizations, or those of the publisher, the editors and the reviewers. Any product that may be evaluated in this article, or claim that may be made by its manufacturer, is not guaranteed or endorsed by the publisher.
